# NetMatchStar: an enhanced Cytoscape network querying app

**DOI:** 10.12688/f1000research.6656.2

**Published:** 2015-11-03

**Authors:** Fabio Rinnone, Giovanni Micale, Vincenzo Bonnici, Gary D. Bader, Dennis Shasha, Alfredo Ferro, Alfredo Pulvirenti, Rosalba Giugno

**Affiliations:** 1 Department of Math and Computer Science, University of Catania, Catania, 95125, Italy; 2 Department of Computer Science, University of Verona, Verona, 37134, Italy; 3 The Donnelly Centre, University of Toronto, Toronto, ON, M5S 3E1, Canada; 4 Department of Computer Science, Courant Institute of Mathematical Sciences, New York University, New York, NY, 10012, USA; 5 Department of Clinical and Experimental Medicine, University of Catania, Catania, 95125, Italy

**Keywords:** network querying, exact graph matching, approximate graph matching, cytoscape app, statistical significance, background network models, randomization, biological network motifs

## Abstract

We present NetMatchStar, a Cytoscape app to find all the occurrences of a query graph in a network and check for its significance as a motif with respect to seven different random models. The query can be uploaded or built from scratch using Cytoscape facilities. The app significantly enhances the previous NetMatch in style, performance and functionality. Notably NetMatchStar allows queries with wildcards.

## Introduction

Biological networks such as protein-protein interaction, transcription regulatory, gene regulatory, and metabolic networks are often referred to as complex systems ^1^. The term complex relates to the existence of non-trivial substructures contained within them. The study of complex systems involves the analysis of the way in which their elements interact rather than only their individual roles. Computationally, such a study entails the ability to query networks to find specific patterns of interactions.

Possible queries might include the identification of positive and negative autoregulation, coherent and incoherent feed forward loops, single-input modules and dense overlapping regulons ^2^ in a given target network *N*. Sub-networks that occur surprisingly often in a network may be preferred by evolution. For that reason, NetMatchStar offers the ability to compute a p-value against null models from seven distinct randomizing methods and suggests the one that shares the network properties of *N* in terms of degree distribution, cluster coefficient and assortativity.

The availability of computational tools for the analysis of biological networks has been helpful in providing novel biological insights on the function of many previously uncharacterized proteins. Several different methods have been developed for this purpose: (i) Network Motif fiding ^3– 12^, network querying ^13– 15^ and network alignment ^16– 21^ algorithms.

Most of the approaches dealing with this kind of graph analysis entail subgraph matching. Such a problem has been widely studied and several methods and systems have been proposed. The approaches can be categorized according to the methodology they use. The first category is the tree search based algorithm. Those methods look for a solution of the problem in a state space by making use of a depth-first approach. Algorithms using such approach include Ullmann ^22^, VF2 ^23^ and the recently introduced RI ^24^. The second category consists of algorithms using Constrained Programming techniques. Such methods aim at filter pairs of nodes which will not be in a matching solution. Many algorithms exploit such approaches ^25– 27^. The last category uses a database approach by exploiting the virtues of indexing ^28– 31^. Such algorithms extract a set of features which define an index of the query that will be used for searching through the target one. The goal is to identify candidate subgraphs in the target one which are possibly isomorphic to the query. NetMatchStar works on Cytoscape 3.2+ and is based on the NetMatch software in ^13^. It deals with both exact queries and approximate ones, in which wildcards are used to match unspecified number of elements.

NetMatchStar integrates the RI algorithm proposed for biological real networks which outperforms other existing algorithms ^24^. RI uses a search strategy based on the topology of the query to effectively filter the space of solutions. We refer to NetMatchStar web page for use cases. For illustration purposes, NetMatchStar has been tested on a biological dataset ^24, 32^ and an overview of its performance concludes the paper.

## Methods

A graph *G* is a pair ( *V*, *E*), where *V* is the set of nodes and *E* ⊆ ( *V* × *V*) is the set of edges.

Using a graph *Q* to query a target network graph *N* means to perform a subgraph isomorphism, which entails finding an injective function that maps each node of *Q* to a unique node of *N* such that nodes and edges labels are preserved. Assessing the statistical significance of *Q* implies a simulation process, where first a set *R* of *r* random graphs are generated according to a specific model. Then the number of occurrences of *Q* in each random graph is counted and a p-value is computed which is defined as the fraction of the *r* graphs where *Q* occurs at least as often as in *N*. The lower the p-value is, the more significant *Q* is as a motif. The significance of *Q* can also be evaluated through the z-score, which is defined as the difference between the number of occurrences of *Q* in *N* and the average number of occurrences of *Q* in the *r* random graphs, divided by the standard deviation of the frequencies of *Q* in *R*. A strongly positive value of the z-score means that *Q* is significant as a motif.

### Exact querying

A simple enumeration algorithm to find *Q* in *N* generates all possible maps between the nodes of the two graphs and checks whether any generated map is a subgraph isomorphism. The common aim of existing algorithms is to discover unsuccessful mappings as early as possible and to filter them away ^22^. NetMatchStar uses the algorithm RI proposed in ^24^, whose efficiency is mainly due to the choice of a search strategy, i.e. the ordering with which query nodes are mapped. For example, a variable ordering may begin with a query node having the highest degree or having the most uncommon label in the target graph. The variable ordering of RI is based only on the query graph topology. Roughly, the chosen order creates constraints as early as possible in the matching phase. The nodes having high valence and that are highly connected with nodes previously present in the ordering tend to come early in the variable-ordering. The aim of RI is to avoid costly pruning techniques by finding a static search strategy such that the number of constraints that are verifiable from a partial solution are maximized.

### Approximate querying

Approximate queries are graphs containing wildcard structures. They may contain nodes and edges which can match any value of node or edge labels in the network and approximate paths constrained in length to be less than or greater than *m*, where *m* is a positive integer. NetMatchStar first matches all the specified subparts of the queries exactly and then joins the matches by network traversal. The network traversal phase checks that all traversed paths satisfy the query path constraints.

### Random model generation

In NetMatchStar, the user can choose among seven different generative models to compute the statistical significance of a motif. In all cases, except for the shuffling model, the simulation starts with the generation of a network with | *V*| nodes having the same labels as the target network *N* and no edges. Then, new edges between existing nodes are added until we obtain a network with | *V*| nodes and | *E*| edges, just like *N*. In the following, we briefly describe each random model.

***Shuffling model.*** In the shuffling model ^33^ an existing network is "rewired" by repeatedly swapping the destinations of two randomly chosen edges, where possible. The result is a graph with the same degree distribution of the original network.

***Erdos-Renyi model.*** The Erdos-Renyi (ER) model ^34^ corresponds to a graph where two nodes connect each other randomly and independently. There are two variants of the ER model. In the *G*(| *V*|, | *E*|) model the algorithm randomly creates a network uniformly over all networks that have | *V*| nodes and | *E*| edges. In the *G*(| *V*|, *p*) model, edges between nodes are independently created with a user-defined probability *p*. NetMatchStar implements the *G*(| *V*|, *p*) variant of the ER model.

***Watts-Strogatz model.*** The Watts–Strogatz model ^35^ produces graphs characterized by the small-world property, where most nodes can be reached from every other by a small number of hops, when there is no direct link between them. The model works in two phases. In the first one a lattice of | *V*| nodes is created where each edge is connected to *d* neighbors on its left and *d* neighbors on its right. Then, edges are randomly shuffled with rewiring probability *β*. Low values of *β* produce a quasi-regular graphs, where nodes have approximately the same degree, while high values of *β* produce networks which are very close to the ER model.

***Barabasi-Albert model.*** Also known as the preferential attachment model, this model ^36^ creates graphs where the more connected a node is, the more likely it creates new links. Graphs generated with BA model are scale-free, meaning that the degree distribution follows a power law, with a few high-degree nodes and many low-degree nodes. The BA model starts with the creation of a complete initial seed network of *k* nodes. The remaining | *V*| – *k* nodes are added one at a time. Each new node is attached to *d* existing nodes, such that the probability of selecting an existing node *u* is proportional to the degree of *u*.

***Geometric model.*** The geometric model ^37^ describes graphs in which the information about the location of nodes in the space determines the topology and might be useful to represent spatially oriented networks (e.g. transportation and neuronal networks). In the geometric model each node is represented as a point in a *d*-space. An edge between two nodes exists if the distance between corresponding points is within a threshold *r*.

***Forest-Fire model.*** In the Forest-Fire (FF) model ^38^, a new node *v* attaches to the network by iteratively exploring existing edges starting from one or more anchor nodes, called ambassadors, which are chosen randomly. At each step of the exploration, *v* creates out-links with newly discovered nodes with a forward probability *p* and in-links with a backward probability *r*, and continues exploration from those nodes. The FF model describes time-evolving networks where the number of edges grows super-linearly in the number of nodes and the distance between nodes shrinks as new nodes arrives.

***Duplication model.*** In the duplication model ^39^ the duplication of the information is considered as a dominant evolutionary force for the growth of a network, such as in many biological networks. At each step of the duplication model a random node *u* is selected. Then, a new node *v* is created and connected to neighboring nodes of *u* with probability *p*. The lower is *p*, the more divergent is *v* as a copy of *u*.

### Implementation

The NetMatchStar Cytoscape App has been developed in Java 7 on top of the Cytoscape 3.2 API. The software is composed by a core module, which implements basic algorithms and data structures, plus a user interface module that integrates the analyses into the Cytoscape interface. The core module provides data representations, graph analysis (i.e. graph matching and motif searching) and two different types of attribute comparator that differentiate in exact and approximate comparison. The CyNetworks are converted into graph structures to optimize the graph traversal procedures. The user interface is designed by following the Model-View-Controller architectural pattern. The Model component adds up result data representations to the functionality provided by the software’s core module. The View component implements the graphical panels of the interface. The main panel of the app adds up, as a further tab, to the Control Panel of the Cytoscape interface. This integrates the graphical panels where the user can select the networks to be processed, the parameters of the analysis, and the results. The Control component ensures the communication between the Model and the View by implementing the set of tasks performed by NetMatchStar. This component is developed by following the Cytoscape 3.1 app guidelines, such that every task is implemented as a Cytoscape Task Java class.

### Operation

**Figure 1.**
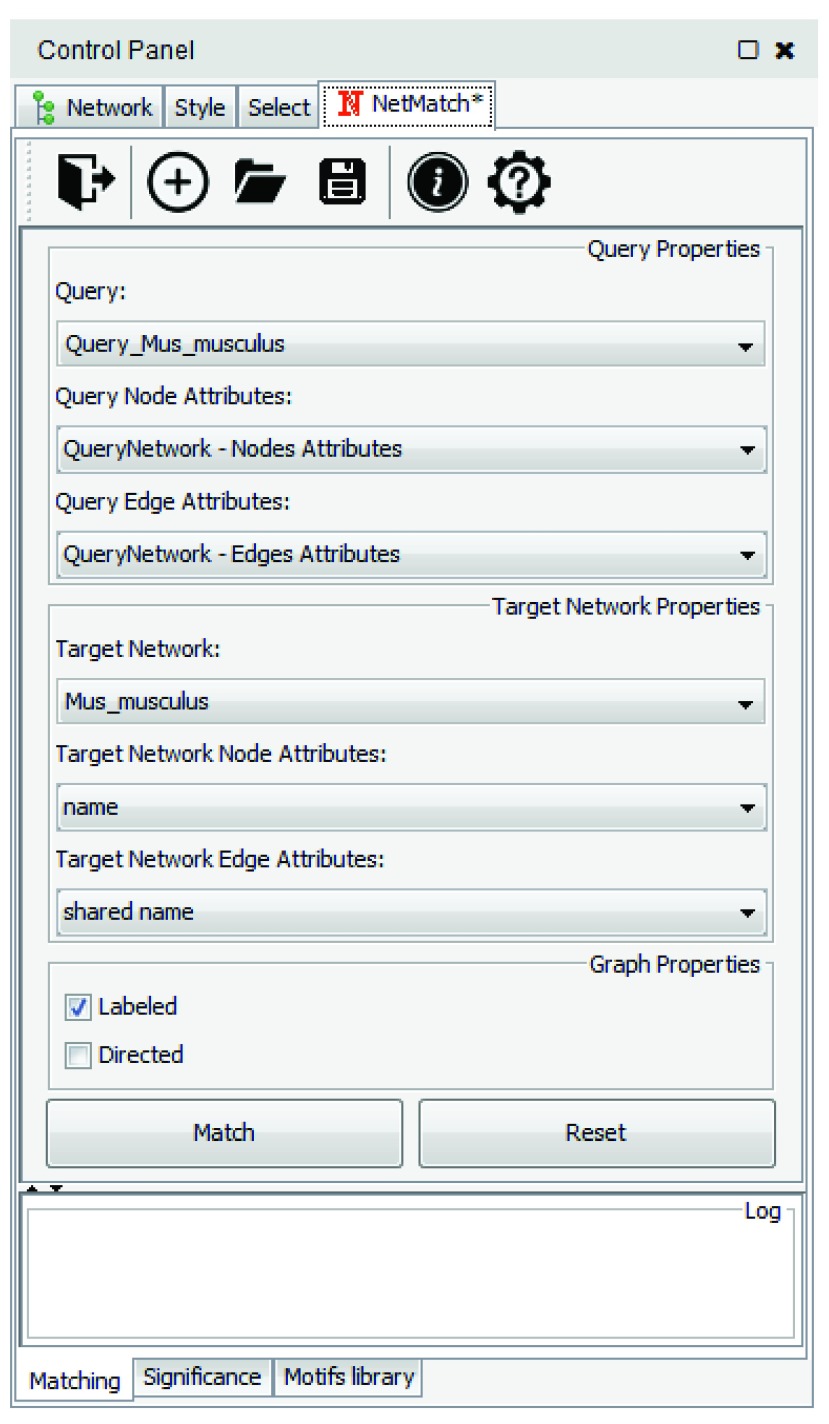
"Matching" panel in NetMatchStar. In this example, the network of Figure 4 has been provided as query, while the *Mus musculus* network provided in 24 has been chosen as target graph.

**Figure 2.**
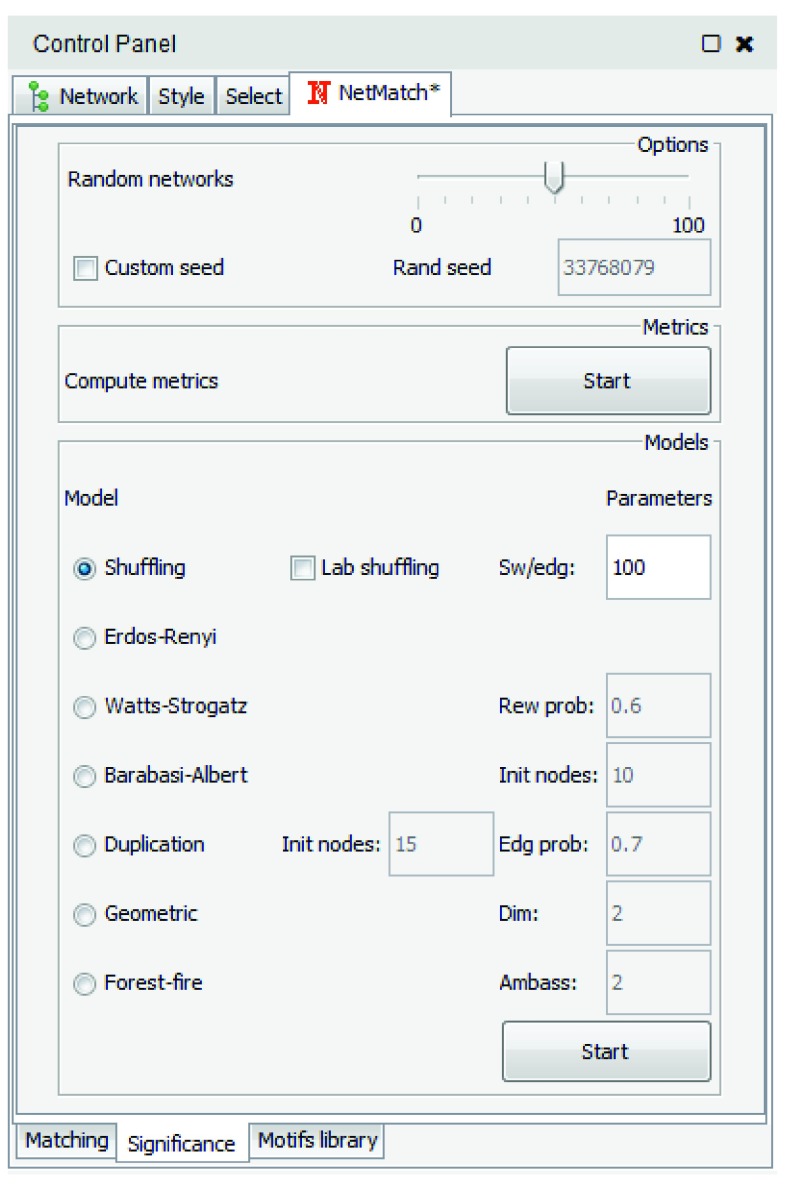
“Significance” panel in NetMatchStar.

**Figure 3.**
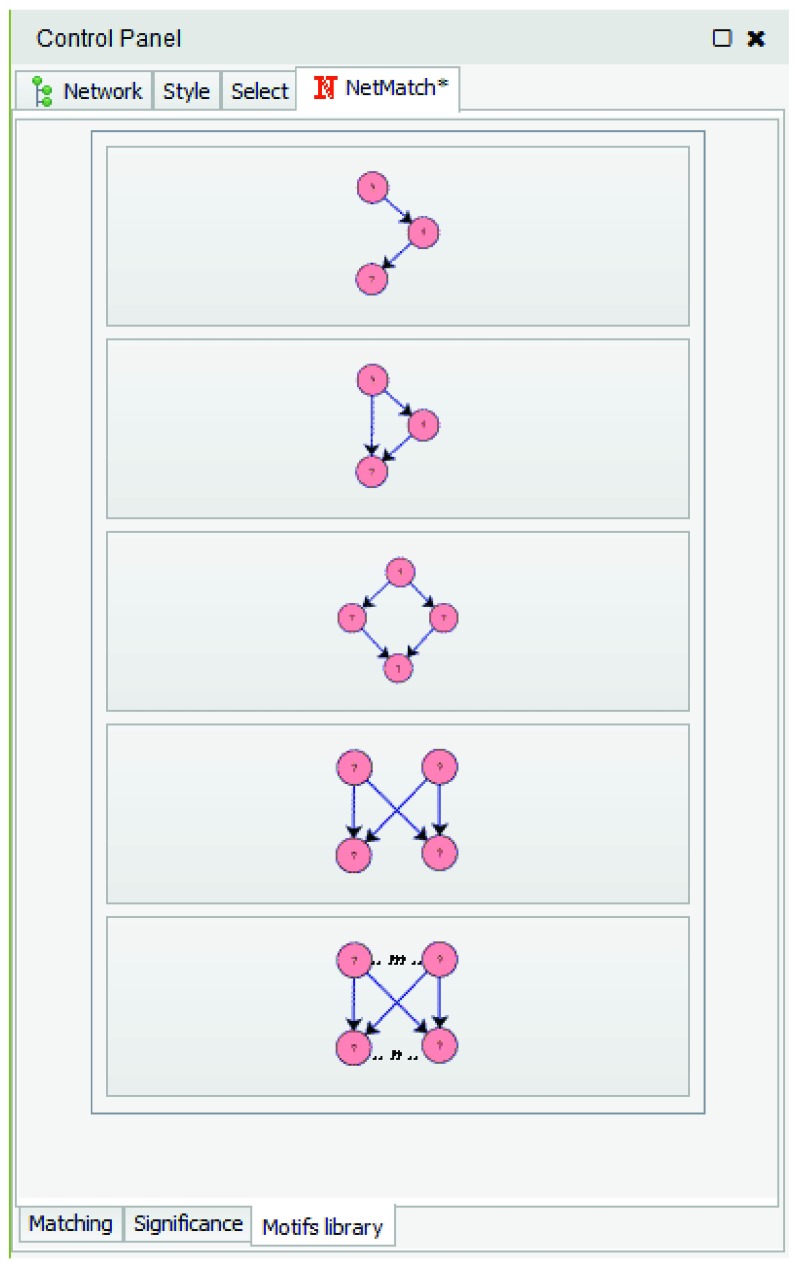
"Motif library" panel in NetMatchStar.

The main frame of NetMatchStar contains three tabbed panels:

- "Matching" panel ( Figure 1), to specify the target and the query graphs and run the matching task;
- “Significance” panel ( Figure 2), for the statistical significance of the query as a motif according to a specific random model;
- "Motif library" panel ( Figure 3), which contains a set of predefined queries for the matching task.

In the following subsections, we will describe all the required steps for the matching and motif verification of a query graph in a target network.

### Loading input data

Query and network graphs can be uploaded in NetMatch-Star, by clicking on the folder icon in the toolbar of "Matching" panel ( Figure 1). Each uploaded network will be added to the Network list of Cytoscape. In the drop-down lists of "Network Properties" and "Query Properties" section, the user can select one of the uploaded networks as a query or target network for the matching and statistical significance tasks. Likewise, the user may upload node and edge labels as Cytoscape attributes and link them to the nodes and edges of the target network and query graph.

### Drawing queries

Instead of loading an existing network, the user can create a query from scratch or by starting from a pre-defined set of queries.

**Figure 4.**
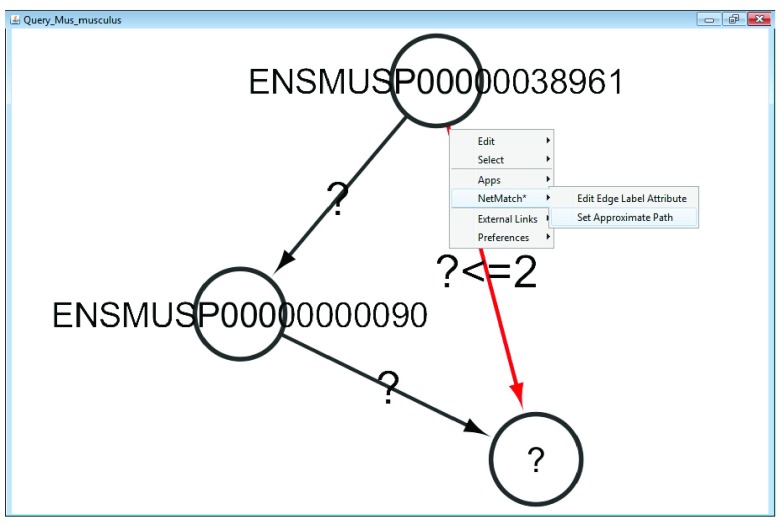
Panel for the creation of a query network in NetMatchStar. In this example, an approximate query with 3 nodes and 3 edges has been created, where 2 nodes have a specific label and one edge represents an approximate path of length at most 2 (’?<=2’). The remaining elements of the graph have an unspecified label (’?’). By selecting an edge and right-clicking, a menu will be shown for changing its label or set the approximate path.

To create a new query, the user must click on the "plus" icon of "Matching panel" ( Figure 1). A new panel for the creation of a new network will be opened ( Figure 4). A right click on the panel will open the standard Cytoscape menu to add, edit or remove elements of the graph. Such a menu also includes the "NetMatchStar" menu item, which lets the user change the label of a node or edge and set a path between two nodes. By default, newly added nodes and edges will be labeled with the wildcard "?", corresponding to a node or a direct link between nodes with unspecified label. Any other character will be associated to a specific label. Paths between two nodes *i* and *j* are defined as special attributes for the edge ( *i*, *j*). The length of a path is specified by an expression of the form *aopb*, where *a* and *b* are two integers (or the wildcard "?") and *op* is one of <, ≤, ≥, >, =. The "?" character is used to leave the minimum or maximum length of the path unspecified. For instance, the expression ”? ≤ 2” means that the corresponding path must have at most length 2, while ”? *>* 3” corresponds to a path of length greater than 3. A query with a "?" character in at least a node and/or edge is an approximate query for NetMatchStar.

By clicking on "Save" button on panel, the user can store the query graph created from scratch on disk as text files in a .SIF format with nodes and edges attribute files with extensions respectively .NA and .EA.

The pre-defined set of queries includes small topologies which have been identified as motifs in many real networks ^2^, such as feed-forward loops, diamonds, single-input modules and dense overlapping regulons. Figure 3 shows all the pre-defined queries that can be selected from the "Motifs library" tabbed panel. They are drown as directed graphs but can be used to query both directed and undirected networks. By clicking on one of these topologies, the user can visualize the query and modify it, as previously described, i.e. adding new nodes/edges, changing node/edge labels and setting paths between nodes. Modifying the pre-defined query does not change the original “library” entry, but only a copy of it.

### Evaluating the Statistical Significance of motifs

The “Significance” panel ( Figure 2) contains all the parameters for the evaluation of the statistical significance of a motif subnetwork. It consists of three subpanels. In the top subpanel the user can choose the number of random graphs to generate for the statistical test (between 0 and 100) and the seed for generating pseudorandom numbers. In the middle subpanel the user can compute a set of metrics for the target graph and sample random graphs, one for each model. Metrics include the average degree, the average clustering coefficient and the assortativity index. At the end of the computation, the resulting values are shown in a separate window. Usually, values of these metrics coherent with the one of the input network can suggest to the user which random model best describes the features of the input network.

The bottom subpanel let the user choose a random model and set its parameters (if any). In “Shuffling” model, “Lab shuffling” option can be selected for enabling shuffling also on node and edge labels (if present), while “sw/edg” denotes the number of successful swaps per edges. The “Erdos-Renyi” model has no parameters. In “Watts-Strogatz”, “Rew prob” is the probability of rewiring *β*. The "Barabasi-Albert” model defines “Init nodes”, the number of initial nodes in the complete seed network. The “Duplication” model has two parameters: “Init nodes”, the number of nodes in the initial seed network, and “Edg prob”, the edge duplication probability. In the “Geometric” model, parameter “Dim” denotes the dimension of the space where points are placed. Finally, “Forest-fire” contains parameter “Ambass”, that is the number of ambassadors nodes. For each model, all the remaining parameters are estimated based on the number of nodes and edges of the target network.

### Managing results

Once a target network and a query has been provided in the "Matching" panel ( Figure 1), the user can either look for all occurrences of the query within the input graph or check if the query is a motif or not.

**Figure 5.**
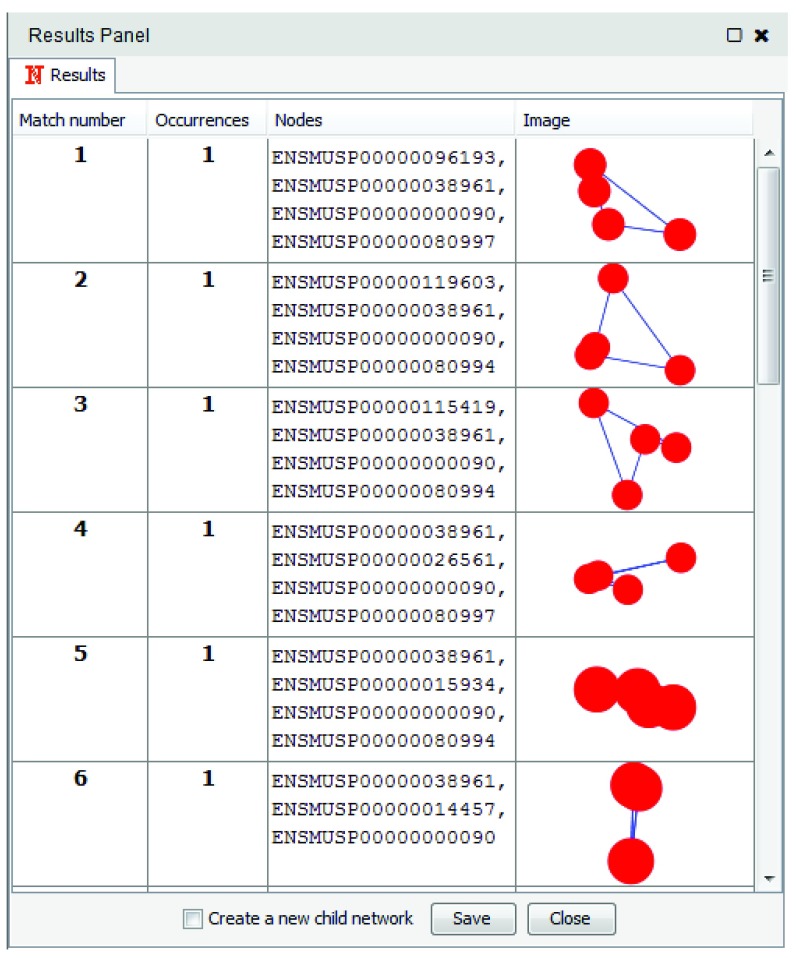
NetMatchStar result table for the matching between the query and the target networks of Figure 1.

In the first case, the user must click on the "Match" button in the "Matching" panel ( Figure 1). Once the matching task has been completed, a table with all the occurrences of the query in the target will be shown as a tabbed panel in the "Result Panel" of Cytoscape ( Figure 5) and the input graph will be visualized. For each occurrence, NetMatch-Star reports its nodes and an image depicting its topology. By selecting a row in the table, the user can visualize the corresponding occurrence in the target network. If the option "Create a new child network" is disabled, nodes of the occurrence will be highlighted in yellow within the input network, otherwise the occurrence will be visualized in a separate window. By clicking on "Save" button on result panel, the user can store the results as text file.

Recalling that the nodes of the network are not uniquely labeled and thus the query may have different matches, to check if a query is a motif, the user must click on one of the "Start" buttons of the “Significance” panel ( Figure 2), depending on the random model that has been chosen to perform the significance test. When the simulation ends, a new window will appear with the following measures: the number of query occurrences in the real network, the mean and the standard deviation of the number of query occurrences in the random networks, the p-value and the z-score. The statistics of the test will be also reported on the “Log” panel located at the bottom of the “Matching” panel ( Figure 1) and they can be consulted anytime.

## Results

We evaluated the performance of NetMatchStar on the biological networks provided in ^24^ and compared it to the original NetMatch, developed for Cytoscape 2.8.

In Cytoscape others software are available for network motifs search. CytoKavosk ^40^ is based on counting all k-size sub-graphs of a given network graph, while GraMoFoNe ^41^ emulates the interface of NetMatchStar by allowing users to define a query and finding all occurrences similar to the query, with respect to node and edge deletions and node similarities. NetMatchStar contains predefined motif structures, checks the significance of a motif with respect to seven different random models and allows user to draw queries containing wildcards and manage the approximation they need.

**Figure 6.**
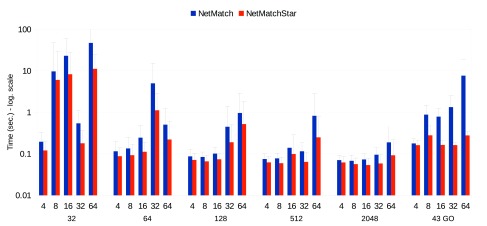
Query execution time on PPI networks.

Figure 6 depicts the evaluation of NetMatchStar on three protein-protein interaction networks: *Mus musculus*, *Homo sapiens* and *Danio rerio*. They are large dense graphs. We randomly labeled networks with 32, 64, 128, 512 and 2048 synthetic labels and with 43 real labels corresponding to the Gene Ontology (GO) classes of the proteins (i.e. the nodes in the network). We used queries extracted from the networks by varying the number of nodes from 4, 8, 16, 32, and 64 and density from low to high (up the 90% of edges among nodes are present).

**Figure 7.**
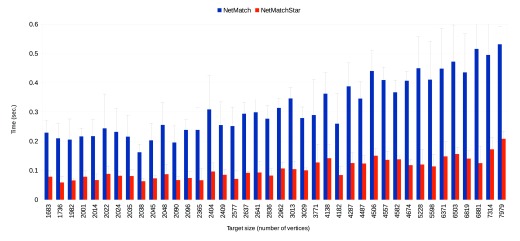
Query execution time on 3d-proteins.

Figure 7 evaluates NetMatchStar on protein back-bones graphs. They are large sparse graphs. The original labels are maintained since they are not unique (i.e., atoms names).

**Figure 8.**
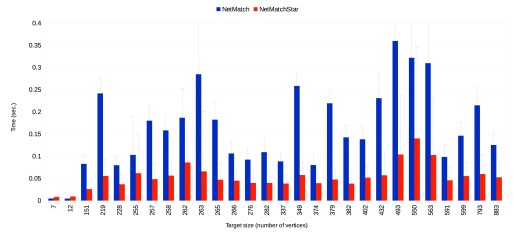
Query execution time on protein contact maps.

Figure 8 evaluates NetMatchStar on contact map graphs. They are dense medium graphs. The original labels are maintained since they are not unique (i.e., amino acids).

**Figure 9.**
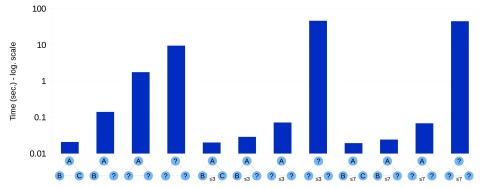
Feed forward loop with wildcards running time on *Mus musculus* with 512 labels.

Figure 9 reports the querying performance of feed forward loop topology on *Mus musculus* with 512 labels. Queries are run exactly and approximated by unspecifying one, two and all node labels and replacing one edge with an approximate path constrained to less than 3 and 7 edges.

**Figure 10.**
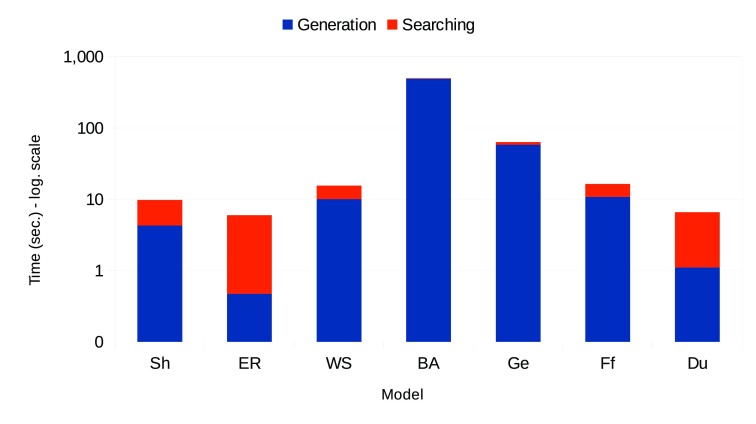
Running times for generating random networks and searching the feed forward loop on *Mus musculus* with no labels according to Shuffling (Sh), Erdos-Renyi (ER), Watts-Strogatz (WS), Barabasi-Albert (BA), Geometric (Ge), Forest Fire (Ff) and Duplication (Du) models.

**Figure 11.**
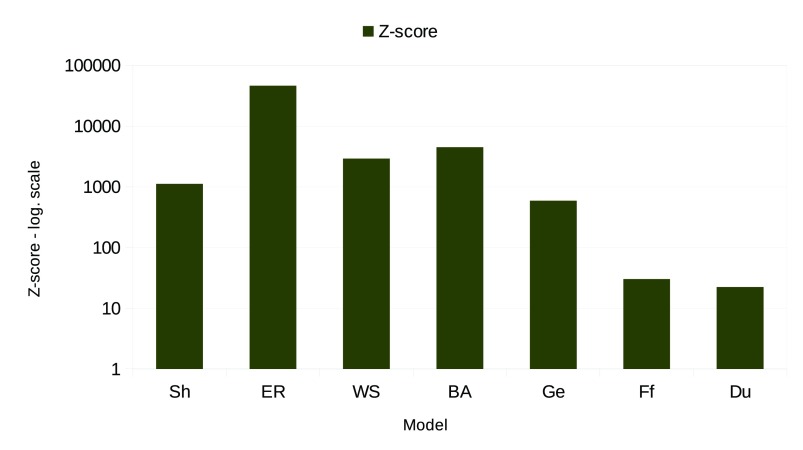
Z-scores for the feed forward loop on *Mus musculus* with no labels according to Shuffling (Sh), Erdos-Renyi (ER), Watts-Strogatz (WS), Barabasi-Albert (BA), Geometric (Ge), Forest Fire (Ff) and Duplication (Du) models.

Finally, for those queries we verified their statistical significance by using all random models ( Figure 10) and we measured the average time required for generating random networks and searching the queries ( Figure 11).

## Summary

This paper presented the biological network querying system NetMatchStar for Cytoscape 3.2.1. NetMatchStar improves upon its predecessor NetMatch in usability and performances. Moreover, it allows a comprehensive evaluation of statistical query significance. Future work includes semantic and ontological similarity search.

## Software availability

This section will be generated by the Editorial Office before publication. Authors are asked to provide some initial information to assist the Editorial Office, as detailed below.

### Software available from

http://apps.cytoscape.org/apps/netmatchstar,

http://alpha.dmi.unict.it/netmatchstar/

### Latest source code

https://github.com/fabiorinnone/NetMatchStar/tree/v3.1

### Link to source code as at time of publication

http://dx.doi.org/10.5281/zenodo.19045 ^42^

### License

Creative Commons Attribution-NonCommercial-ShareAlike 3.0 License.
