# Supplementary material for: NetMatchStar: an enhanced Cytoscape network querying app
Source: F1000Res. 2015 Nov 3;4:479. Originally published 2015 Aug 5. [Version 2] doi: 10.12688/f1000research.6656.2 (PMC4642848; doi:10.12688/f1000research.6656.2)
Supplement: Peer review history

# Peer review: Referee response for version 1

**DOI:** 10.5256/f1000research.7150.r10336

**Published:** 2015-09-21

**Authors:** Shaillay Dogra

**Affiliations:** 1 Vishuo BioMedical Pte Ltd, Singapore, Singapore

Algorithmically, is a useful utility that has been implemented by the authors. Bringing out its multiple use-case scenarios in biological setting with interpretations for example will help novice users adapt this more widely. Biological explanations of network interpretations will strengthen understanding and usage. Also, there are 7 different algorithms implemented. In which kind of data or user-problem which algorithm is recommended, according to authors or empirical observations?

Minor points:

- "Algorithms using such a technique include.." -- kindly mention here itself
- "the RI algorithm proposed for biological real networks which outperforms other existing algorithms.."  -- please elaborate on this RI algorithm
- "Values of these metrics can suggest to the user which random model" - can the authors recommend some approximation of good/bad values?

I have read this submission. I believe that I have an appropriate level of expertise to confirm that it is of an acceptable scientific standard.

# Peer review: Referee response for version 1

**DOI:** 10.5256/f1000research.7150.r10314

**Published:** 2015-09-15

**Authors:** Xifeng Yan

**Affiliations:** 1 Computer Science Department, University of California, Santa Barbara, Santa Barbara, CA, USA

NetMatchStar improves the previous NetMatch tool, a Cytoscape app developed by the authors. It has more functions (e.g., supporting approximate querying with wild cards) and runs faster. It shall be interesting to users who are looking for tools that are able to find all the occurrences of a query graph in a network and check for its significance under different null models. The manuscript has detailed description and evaluation results.

I have read this submission. I believe that I have an appropriate level of expertise to confirm that it is of an acceptable scientific standard.
